# Preclinical Safety Assessment of an Ayurvedic Dentifrice: Acute and 28-Day Oral Toxicity Studies in Wistar Albino Rats

**DOI:** 10.1155/jt/1379571

**Published:** 2025-08-26

**Authors:** Delfin Lovelina Francis, Saravanan Sampoornam Pape Reddy, Balaji Manohar, Shaili Pradhan

**Affiliations:** ^1^Department of Public Health Dentistry, Saveetha Dental College & Hospitals, Saveetha University, Saveetha Institute of Medical and Technical Sciences (SIMATS), Chennai, Tamil Nadu 600077, India; ^2^Department of Periodontology, Army Dental Corps, Delhi 110010, India; ^3^Department of Periodontology, Geetanjali Dental and Research Institute, Geetanjali University, Udaipur, Rajasthan 313001, India; ^4^Department of Periodontology and Oral Implantology, Kathmandu Medical College Public Limited, Kathmandu 44600, Nepal

**Keywords:** Ayurvedic, dentifrices, drug evaluation studies, medicine, preclinical

## Abstract

**Background:** Herbal formulations with antimicrobial and anti-inflammatory properties are commonly used in traditional medicine for oral hygiene. Despite growing popularity, little or no research exists on the safety profile of these products to confirm their long-term safety. An ayurvedic dentifrice formulation was analyzed in Wistar albino rats for acute and subacute oral toxicity, with emphasis on the toxic effects of the product on physiological, hematological, biochemical, and histopathological parameters.

**Methods:** Wistar rats (*n* = 6) were administered a single dose of the test formulation above 2000 mg/kg body weight and were kept on a 14-day observation period for acute toxicity. Subacute toxicity was induced by daily oral administration of 300, 600, and 1000 mg/kg of the test formulation to male (*n* = 30) and female (*n* = 30) rats for 28 days. A 14-day recovery period in the high-dose group was then carried out. Clinical signs, including mortality, food and water intake, body weight, hematological and biochemical parameters, vital organ weights, and histopathological alterations, were assessed.

**Results:** Acute and subacute toxicity studies did not reveal any mortality or significant clinical signs. At 2000 mg/kg body weight, the hepatic changes were minor and reversible. The no-observed-adverse-effect level (NOAEL) was determined to be 1000 mg/kg body weight, as there were no significant changes in biochemical, hematological, and histopathological parameters until this dose.

**Conclusions:** In Wistar albino rats, the tested Ayurvedic dentifrice formulation tapered with quiescent toxicity can be considered safe for oral use. Long-term studies in the form of clinical trials are recommended for human use.

## 1. Introduction

Oral health is a crucial aspect of overall health, with dental caries and periodontal diseases ranking among the foremost global issues [1]. Although conventional fluoride-based dentifrices are commonly used, there is increasing interest in the use of plant-derived dentifrices, as they possess antimicrobial, anti-inflammatory, and antioxidant properties [2]. Various herbal components in oral care, including *Salvadora persica* (miswak) [3], *Terminalia chebula* [4], *Zingiber officinale* (ginger) [5], *Syzygium aromaticum* (clove) [6], and *Elettaria cardamomum* (cardamom) [7], have been used in complementary and alternative medicine (CAM), with studies demonstrating their potential for antimicrobial protection [8]. However, systematic toxicological assessments of herbal dentifrices are currently insufficient, implying that, thorough safety evaluations are required [9]. The scientific evidence supporting the safety of herbal dentifrices is still limited, despite their increasing commercial availability [10]. These formulations must undergo rigorous biocompatibility and systemic safety testing. The regulations of the Organization for Economic Co-operation and Development (OECD) states that, after repeated exposure, adverse effects can occur; thus, both acute and subacute toxicity studies should be performed. These experiments examine critical physiological endpoints, such as hematological and biochemical indices, organ function, and histopathology, which ultimately establishes the formulation of the no-observed-adverse-effect level (NOAEL) [11]. This study evaluated the safety profile of an Ayurvedic dentifrice formulation in Wistar albino rats by analyzing clinical signs, hematology, biochemistry, and organ histopathology after acute and subacute administration.

## 2. Materials and Methods

The study was performed according to the ARRIVE 2.0 guidelines [12]. All procedures were performed in compliance with the guidelines issued by the Committee for the Purpose of Control and Supervision of Experiments on Animals (CPCSEA) for laboratory animal facilities in India, and the protocol was approved by the Animal Ethical Board under letter no. CKL/TOX/IAEC/2021-3/169-November 13, 2021. The test item was an Ayurvedic dentifrice formulation (Composition, Supporting Table 1), and distilled water was used as a vehicle. For the acute toxicity study, six female nulliparous and non-pregnant rats aged 8–12 weeks and weighing 160–220 g were used. For the subacute toxicity assay, 30 male and 30 female nulliparous and non-pregnant Wistar albino rats, weighing 220–360 g (males) and 190–260 g (females), aged 8–12 weeks, were used. The sample size was calculated using G∗Power Version 3.1.9.7 (2020), for effect size (*f*) = 0.25, *α* = 0.05, 1 − *β* = 0.80; the recommended sample size is 10 per group (*n* = 60). Animals were kept in an air-conditioned room with adequate fresh air, a temperature of 22 ± 3°C, relative humidity of 65%–70%, and a 12-hour light and dark cycle. Measurements of relative humidity and temperature were recorded daily. Each animal was housed in a polysulfone cage (dimensions: *L* 300 mm × *W* 170 mm × *H* 140 mm) with pelleted chow and drinking water *ad libitum* with sterile rice husk bedding. The animals were acclimatized to laboratory conditions for 7 days, and clinical signs were observed daily. The veterinary checkup was recorded on day one and day seven during acclimatization. Animals of the same age group, sex, weight, and health status were randomly selected using a random number generator. A stratified randomization technique based on sex for a randomized dosing order and dosing schedule was adhered to. The person administering the test dose (S.S.P.R) and analyzing the data (D.L.F), including histopathological analysis, was blinded.

### 2.1. Test Substance Characterization

The test product utilized was a commercially available Ayurvedic dentifrice (Edinora, Thiruvananthapuram, Kerala, India). The formulation was provided as a prefilled product, which was stored in a sealed container protected from light at room temperature for the duration of the study. The toothpaste was suspended in distilled water at a concentration used for dosing drinks prior to feeding. Heavy metals content was determined using atomic absorption spectrophotometry, and their levels, that is, lead, mercury, arsenic, and cadmium, were found within permissible limits according to AYUSH and WHO standards.

### 2.2. Acute Toxicity Experiments

The acute toxicity protocols involving animals are shown in Table 1. In the sighting trial, dosing was performed sequentially using a minimum interval of 24 h before the next dose was administered to another animal. Five animals were used at each dose level in the main study. One of the five animals, administered a dose of 2000 mg/kg, was from the sighting study, and the remaining four were from other animals. The lethal dose 50% in the oral acute toxicity study (LD50) was determined using the Wistar albino rats according to OECD Guideline 423 (Acute Oral Toxicity—Acute Toxic Class Method). As the toxicological profile of the Ayurvedic dentifrice was not available, a low starting dose (300 mg/kg bwt) was chosen based on OECD guideline 423 for testing acute oral toxicity. As no adverse effects were noted at 300, 2000 mg/kg was selected for the main study, as this is a limit dose for substances expected to have low acute toxicity. The dose escalation for each level in the sighting study was dependent on the occurrence and severity of the toxic signs and the time of onset of the toxic signs from the initial 24 h of the post-administration in accordance with the guidelines of OECD 423. Each animal was observed individually for a minimum duration of the first 30 min following dosing, then at 4-hour intervals throughout the first 24 h, then daily, until a maximum of 14 days. All animals were euthanized by CO^2^ inhalation and subjected to necropsy and histological examination (HPE). A blinded outcome assessment design (B.M) was employed to minimize observer bias. Animals were housed and coded labeled (code not related to group). The observer recording clinical signs, mortality, body weight, feed and water consumption, and performing postmortem was blinded to the treatment groups. Group assignment keys were kept by personnel staff throughout the study period.

The test substance was prepared immediately before administration in the vehicle at the volume required for the dosage (mg/kg body weight). Animals were fasted overnight (with ad libitum access to water) prior to dosing. The test substance was administered as a single dose for each animal, receiving an equivalent of 1 mL per 100 g body weight through a gavage needle, according to the latest body weight. On day one following dosing, all animals were evaluated clinically and assessed for mortality every 30 min for the first 4 hours. Subsequent twice-daily observations and assessments for mortality/morbidity were undertaken over the 14-day period. The clinical examination of animals included assessment not limited to the skin, fur, eyes, mucous membranes, tremors, convulsions, salivation, diarrhea, lethargy, sleep, coma, respiration, somatomotor activity, and behavior. Changes in body weight were calculated just before treatment, before dosing, then every week, and pre-euthanasia. The daily intake of food and water was documented during the study period.

### 2.3. Subacute Toxicity Experiments

After preparation of the test formulation according to the dosage (mg/kg body weight), oral administration to animals by gavage was performed. The subacute toxicity animals were grouped based on Table 2, and this administration procedure was performed for 28 days in a row. The 28-day repeated dose oral toxicity test was performed according to the OECD Test Guideline 407 (Repeated Dose 28-Day Oral Toxicity Study in Rodents) for the systemic toxicity of the test compound after a daily oral administration. Dose levels for the 28-day subacute toxicity study were selected according to the results of the acute study. The maximum dose used was 1000 mg/kg/day, and middle (500 mg/kg/day) and low (250 mg/kg/day) doses were scaled based on the maximum dose and used to investigate potential dose-related effects. Food and water intake per rat were assessed, and the food intake of the recovery group rats was recorded weekly during the post-treatment phase. On day 28, all animals in the test group and all animals in the recovery group on day 42 were subjected to overnight fasting. During fasting hours, ad libitum water was provided. Euthanasia was carried out for Groups I, III, IV, and V on the 28^th^ day and for Groups II and VI on the 42^nd^ day; blood samples were collected through cardiac puncture. Hemoglobin (Hb), leukocyte count (WBC), erythrocyte count (RBC), and platelets were the hematological parameters studied. Biochemical parameters included total protein, albumin, bilirubin (total and direct), glucose, urea, uric acid, creatinine, aspartate aminotransferase (AST), alanine aminotransferase (ALT), sodium, chloride, alkaline phosphatase (ALP), potassium, total cholesterol (TC), HDL cholesterol, calcium, and triglycerides (TGs). After euthanasia, tissue samples from relevant organs, such as the liver, heart, lung, kidney, spleen, stomach, brain, and testis/ovary, were harvested, and 5 μm sections were stained with hematoxylin and eosin (H&E) for HPE. During this study, no changes were made until completion. Data on body weight, food intake, water intake, and hematological and biochemical parameters were statistically analyzed using GraphPad Prism software, Version 7.00, United States. One-way ANOVA was employed to compare between the groups, and Dunnett's post-test was used to compare the variation of different test groups from controls. The control recovery and high-dose recovery groups were analyzed using an unpaired *t*-test. Comparisons and analyses were performed at a 5% significance level. For Groups II and VI (recovery groups), animals were treated with these doses of the tests for a 28-day period and allowed to recover for 14 days in the absence of treatment to determine the reversibility of any adverse effect.

## 3. Results

### 3.1. Acute Toxicity Results

**Figure 1:**
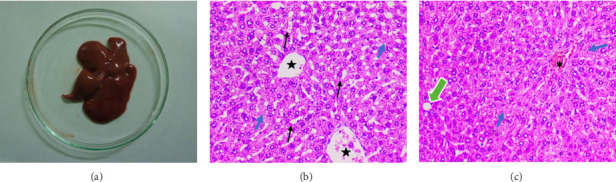
(a) Gross image of the liver from animal treated with 2000 mg/kg body weight of test formulation showing mild enlargement and pale discoloration. (b, c) Histological image of the liver from animal treated with 2000 mg/kg bodyweight of test formulation (H&E 400X). (b) Loss of hepatic architecture with dilatation of the central vein (star) and sinusoids (black arrow) and degenerative changes in the hepatocytes (blue arrow). (c) Central venous congestion (asterisk) and centrilobular hepatocyte degeneration (blue arrow). Mild fat vacuolation was also noted in hepatocytes (green arrow).

The average food intake was similar in the three groups during the 28 days of experimentation. Average daily food consumption in male rats was 19.2 ± 0.9 g (Group I) to 19.6 ± 1.1 g (Group V), and in females 18.5 ± 1.0 g (Group I) to 18.8 ± 1.3 g (Group V). There was no significant difference (*p* > 0.05) in food intake between control and treated groups. These results indicate that, the test compound did not harm the appetite or feeding. No clinical signs of toxicity or mortality were observed in the test group. In addition, there were no behavioral changes in rats during the 14-day observation period. No clinical changes were noticed during the physical examination of the rats. At the given doses, no significant changes were observed in body weight, percentage body weight gain, and food and water consumption during the observation period. Histopathologically, there were no significant changes in animals treated with 300 mg/kg body weight of the test sample. Administration of 2000 mg/kg, however, caused mild hepatomegaly and liver pallor in four out of five treated animals (Figure 1(a)). Histologically, there was disruption of the hepatic architecture evidenced by central vein and sinusoidal dilation, as well as centrilobular degeneration of hepatocytes with mild lipid vacuolation (Figures 1(b) and 1(c)).

### 3.2. Subacute Toxicity Results

None of the subacute doses exhibited clinical signs or preterminal deaths. No statistically significant differences were observed in body weight and percentage body weight gain of either test or recovery groups in either gender, compared to respective control groups, indicating that the treatment did not adversely affect body weight. Except for the first week of intervention, the average weekly food consumption of male and female animals treated by the respective methods was equal to that of the normal controls, as well as during the recovery phase. The differences among treatment animals in the average total weekly amounts of food consumed, were not considered toxicologically significant, since similar differences were noted in the recovery control group, which was present until completion of the study. Thus, it can be deduced that administration of the test formulation resulted in no toxicologically significant alterations in average weekly food and water consumption during both the treatment and recovery periods. There were no statistically significant treatment-related effects altering the hematological parameters in both of the test and recovery groups when compared to the normal control groups. Although a significant decrease in Hb level was observed in male rats in Group VI and female rats in Groups IV and V when compared to their respective control groups (*p* < 0.05, *p* < 0.05, and *p* < 0.01, respectively), the results were still within physiological limits for the species. Although RBC values significantly decreased in females in Group V (*p* < 0.01), during the recovery phase, they reverted to the physiological range in Group VI. The parameters that appeared to deviate in the treated groups returned close to normal during the recovery period, implying that the changes, if any, were transient and reversible with cessation of the test compound. Animals in the recovery groups were observed for an additional 14 days after treatment for the reversibility of any effects observed. The other hematological parameters were within the physiological limits and were comparable to the respective control arms (Table 3). The biochemical parameters of both the test and recovery groups were significantly different from those of the control groups. No treatment-associated toxicologically significant variations were observed in the biochemical metrics in the study animals, and all parameters, including liver enzymes, were normal. There were no significant changes in serum levels of ALT, AST, ALP, and bilirubin associated with toxicological effects, indicating that liver functions were intact in test formulation-treated animals for 28 days. In addition, there were no significant differences in serum glycemic levels and lipid markers in both sexes of rats during the treatment and recovery phases in comparison to the respective control animals. Renal function was evaluated by determining the serum levels of creatinine, urea, and uric acid, where none of the values were significantly different from those of the normal control groups in the low (Group III) and mid (Group IV) dose treatment groups. However, there was a significant (*p* < 0.001) increase in serum urea levels in both male and female rats, while concentrations of creatinine and uric acid were significantly (*p* < 0.01) increased in female rats, when compared with normal control rats at high doses (Group V). Three months after exposure completion, all renal function parameters reverted to normal in both groups and were comparable to those observed in the control groups, further confirming reversibility (Tables 4 and 5). Electrolytes (K^+^, Na^+^, Ca^2+^, and Cl^−^) did not show any toxicologically significant difference between the treatment and recovery groups. In contrast, high-dose delivery of the test formulation (1000 mg/kg body weight) was associated with a greater serum K^+^ level in female rats, than in control subjects during the treatment period, which was not observed after the recovery period. The biochemical parameters therefore revealed that there were no significant changes in the renal function upon oral administration of the test formulation for 28 days at lower (250 mg/kg) and mid (500 mg/kg) doses. At a high dose (1000 mg/kg), however, increased urea and uric acid levels, especially in female rats, may indicate a slight, reversible effect on renal function. The results showed that the test formulation was not harmful for hematological and biochemical parameters in rats at a dose of up to 1000 mg/kg.

**Figure 2:**
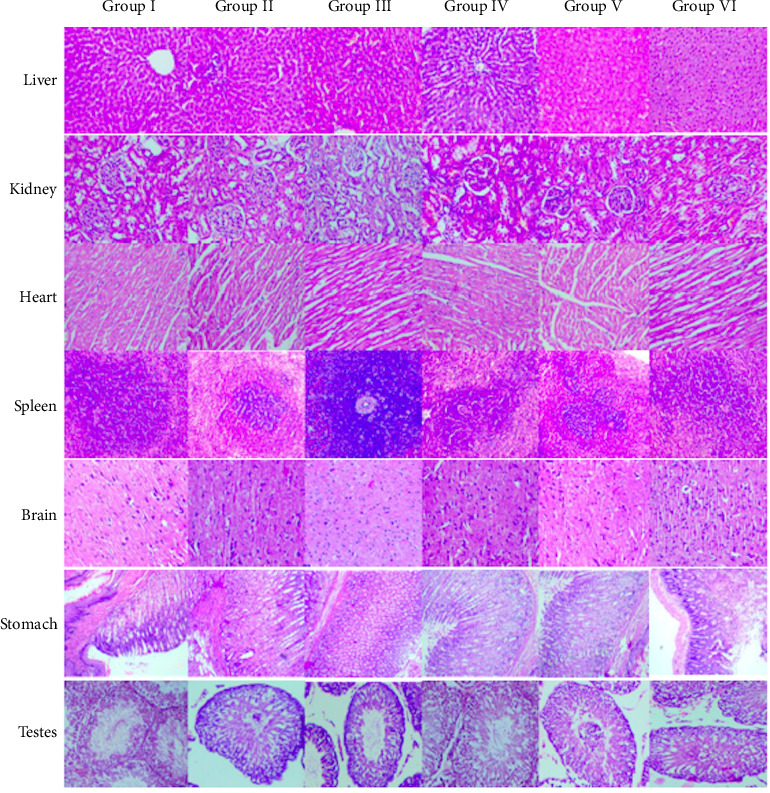
Photomicrographs of tissues of the liver, kidney, heart, spleen, brain, stomach, and testes from male rats of different treatment groups (hematoxylin–eosin–stained microphotograph, 40X), showing normal histological appearance compared to the control group animals. Photomicrograph of internal organ tissues of male rats.

**Figure 3:**
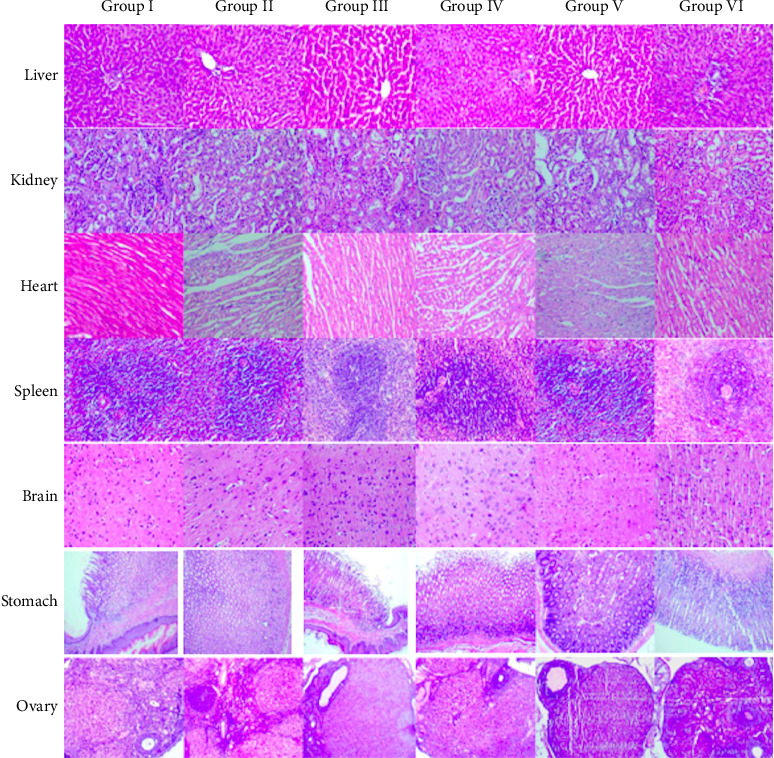
Photomicrographs of tissues of the liver, kidney, heart, spleen, brain, stomach, and ovary from female rats of different treatment groups (hematoxylin–eosin–stained microphotograph, 40X), showing normal histological appearance compared to the control group animals. Photomicrograph of internal organ tissues of female rats.

The gross morphology of the liver, heart, lungs, brain, kidneys, spleen, stomach, and ovaries/testes did not exhibit pathologically significant lesions relative to the control, following administration of the test formulation for 28 days. Furthermore, under HPE, there was no significant treatment-related cellular damage in both test and recovery animals, when compared with corresponding control animals (Figures 2 and 3). During the treatment period, the absolute weight of some organs in animals treated with the test formulation was significantly altered, compared to those in the control group. However, organ relative weights were found to be similar to those of the respective control animals during the treatment and recovery periods. Therefore, it can be said that, the oral delivery of the test formulation for 28 days in Wistar rats, did not cause any significant toxicological effects on the relative weights, gross appearance, and histopathological changes of the internal organs examined, suggesting that the test compound was nontoxic.

## 4. Discussion

This study was undertaken to assess the acute and subacute toxicity of ayurvedic dentifrice preparation (Edinora). The results indicated that the test formulation was safe in Wistar albino rats up to a dose of 1000 mg/kg, without causing any change in body weight, hematological, biochemical, and histopathological parameters. Recent literature states that vigorous testing of plant-based oral care products is essential to understand the safety, efficacy, and relevance of applications to humans [13]. Traditional plant-derived formulations have been used extensively in oral health management, because of their antibacterial [14], anti-inflammatory [15], and antioxidant effect [16]. Recent studies revealed that the periodontal health-promoting potential of ayurvedic dentifrices exhibit an acceptable safety profile [17]. It was observed that plant-based dentifrices containing *S. persica*, *T. chebula*, and *S*. *aromaticum* had antibacterial activity similar to commercially available fluoride-containing formulations without cytotoxicity on gingival fibroblasts [18]. The findings in the current study showed no significant systemic toxicity or organ-specific damage after acute and subacute exposure. In an earlier work, which evaluated a 90-day toxicity study with repeated-doses in rodents for testing an ayurvedic dentifrice and found mild hepatic effects at higher doses (> 2000 mg/kg), but adverse effects were absent at therapeutic doses (500–1000 mg/kg), which was similar to the present study [19]. The mild hepatic changes seen at 2000 mg/kg resolved when exposure had ceased, indicating that, the effects observed were reversible in nature. As hepatotoxicity is unlikely at lower doses, this validates the safety margin of the test formulation.

Blood indices serve as important systemic toxicity indicators [20]. A mild reduction in RBC counts was noted in female rats in the high-dose group (Group V), and other relevant endpoints such as WBC count, platelet count, and Hb levels were comparable between treatment groups in the present study, suggesting a minor hematopoietic effect of the test product. Similarly, the findings from polyherbal formulations reported no detrimental hematological changes observed at therapeutic levels [21]. Biochemical markers for assessing liver and renal functions can provide information about possible toxicities [22]. The present study showed no significant changes in AST, ALT, ALP, bilirubin, or glucose levels, suggesting that the hepatic function was intact even after 28 days of treatment. Similar increases in serum levels of urea and creatinine were observed in a dose-dependent manner among high-dose females (1000 mg/kg body weight), but these changes were reversible in the recovery group. These findings were in agreement with a recent study, which highlighted temporary changes in renal functions due to short-term exposure to herbal mouth rinse, which had reversed promptly [23]. That reversibility of these data implies physiological flexibility, rather than nephrotoxicity [24]. The histopathological analysis of internal organs in this study did not reveal any significant microscopic changes except for single-cell pale foci in the liver of high-dose group rats, which was considered a notable observation. Another study found that, even supratherapeutic levels of a neem-based dentifrice produced mild reversible liver changes [25].

This study showed that, at therapeutic concentrations, the test formulation is nontoxic, suggesting its potential for human applications. Fluoride has been considered the gold standard for caries prevention for many years [26]. It was shown that the herbal formulations were equally effective against oral infections when compared to fluoride-based dentifrices, but did not elicit irritation of mucosal tissues or allergic reactions [27]. Clearly, herbal constituents typically used for medicinal purposes, such as *S. persica* and its derivatives, such as *T*. *chebula* and *S*. *aromaticum*, are safe [28] and are in agreement with this study data. The individual toxicity and therapeutic effects of the various active components present in our evaluated formulation have been previously studied [29, 30]. According to several studies, *S. persica* (miswak) has anti-inflammatory, antibacterial, and antioxidant effects and does not exhibit systemic toxicity in conventional doses [31, 32]. *Z. officinale* (ginger) and *S. aromaticum* (clove) have remarkable antiplaque and anticariogenic potential without showing negative effects on oral health formulations [33, 34]. The effects of virgin coconut oil and cardamom have been proven to reduce the growth of some bacteria that become attached to the teeth and have the potential to cause gingival inflammation as well as increase an unfavorable oral microbiota [35, 36]. No significant and individual component adverse effects were observed, confirming that they are safe, and thus, these compounds can be incorporated in dentifrice preparation. Significant elevation of urea and uric acid at 1000 mg/kg/day, especially in females, was, however, observed but was within the known physiological ranges of urea and uric acid in Wistar rats and was not accompanied by histopathological lesions in the kidney or toxicologically related clinical signs. Furthermore, these alterations were absent during the 14-day recovery, indicating the transient, nondetrimental nature of the effect. Accordingly, it was deemed that, the 1000 mg/kg/day dose was the NOAEL for the current study conditions on the basis of all toxicological findings, such as clinical signs, hematology, serum biochemistry, organ weights, and HPE.

Although the safety of this ayurvedic dentifrice composition (Edinora) has been widely substantiated, there are some limitations that should be taken into consideration. It clearly signified the need to evaluate the chronic exposure (90-day or 6-month toxicity studies), as long-term exposure was not evaluated in this 28-day subacute toxicity study. The hematological, biochemical, and histological parameters were assessed only at a single point of time. The present study did not perform studies of reproductive and immunological changes. The study was presented in descriptive histology and did not assess any objective histopathological scoring methods. The study did not include assessment of biomarkers, gene profiling, and molecular analysis; however, since the study was performed on animals, interspecies differences are a possibility. The study did not compare the data with any other commercially available herbal products, as well as did not assess the herbal drug interactions. Data from animal models with outstanding contributions, will be limited, in how well they can be translated to humans, leading to clinical trials evaluating the safety and efficacy of these interventions in human cohorts. Future studies should address long-term safety, multicenter clinical trials, and comparative effectiveness to conventional fluoride formulations. In addition, plant-molecular approaches that study possible pharmacokinetic interactions may provide better insight into metabolism and concerns on systemic absorption.

## 5. Conclusions

An acute toxicity study of the plant-based dentifrice formulation (Edinora) was performed on Wistar albino rats and showed that no clinical symptoms/death occurred at doses of 2000 mg/kg body weight, although slight hepatic changes were observed at this high level. In subacute toxicity screening at 28 days with a subsequent 14-day recovery period at doses of up to the level of 1000 mg/kg, no significant test formulation-related changes were detected in body weight, food or water intake, or hematological and biochemical parameters. The multi-organ weight, gross pathology, and histopathology were unaffected in a toxicologically meaningful manner. The NOAEL for the formulation was found to be 1000 mg/kg body weight, which shows that the formulation is safe for oral administration in Wistar albino rats. The results demonstrate compelling preclinical safeguards and illustrate that, at therapeutic concentrations, the tested ayurvedic dentifrice formulation is safe for intraoral use and devoid of toxicity. These results consistently emphasize the role of herbal oral care products as safe and effective alternatives to conventional formulations. As a result, further studies, such as chronic toxicity studies in animal models, as well as stringent human clinical trials, are needed to evaluate the long-term safety and benefits to humans along with necessary regulatory approval.

## Figures and Tables

**Table 1:** Study groups for assessment of acute toxicity.

Study particulars	Group	Dose
Sighting study	Group I	One animal was administered a single dose of Ayurvedic dentifrice at 300 mg/kg body weight orally.
	Group II	One animal was administered a single dose of Ayurvedic dentifrice at 2000 mg/kg body weight orally.

Main study	Group III	Four animals were administered a single dose of Ayurvedic dentifrice at 2000 mg/kg body weight orally.

**Table 2:** Study groups for assessment of subacute toxicity.

Groups	No. of rats		Dose
	Male	Female	
I. Normal control	5	5	Single daily dose of distilled water (vehicle) 1 mL/100 g body weight, orally
II. Control recovery	5	5	Single daily dose of distilled water (vehicle) 1 mL/100 g body weight, orally
III. Low dose	5	5	Single daily dose Ayurvedic dentifrice (250 mg/kg body weight), orally
IV. Mid dose	5	5	Single daily dose of Ayurvedic dentifrice (500 mg/kg body weight), orally
V. High dose	5	5	Single daily dose of Ayurvedic dentifrice (1000 mg/kg body weight), orally
VI. High-dose recovery	5	5	Single daily dose of Ayurvedic dentifrice (1000 mg/kg body weight), orally

**Table 3:** Summary of hematological parameters during the treatment period.

Groups	WBC (10^6^ cells/μL)	Hb (g/dL)	RBC (10^3^ cells/μL)	Platelet (10^3^ cells/μL)
Wistar albino rats (males)				
I	5.92 ± 1.549	12.42 ± 0.305	6.392 ± 0.328	772.4 ± 92.28
III	4.52 ± 0.676	12.6 ± 0.707	6.75 ± 0.203	798.6 ± 14.41
IV	4.04 ± 1.075	11.96 ± 0.208	6.292 ± 0.116	751.8 ± 6.414
V	3.2 ± 0.779	12.4 ± 0.363	6.644 ± 0.135	826.6 ± 28.732
II	5.9 ± 0.906	16.38 ± 0.949	7.512 ± 0.052	796.6 ± 40.008
VI	5.06 ± 0.864	12.56 ± 1.161^∗^	6.22 ± 0.682	679.4 ± 35.91
Wistar albino rats (females)				
I	5.08 ± 1.078	13.04 ± 0.102	6.46 ± 0.046	792 ± 37.32
III	3.06 ± 0.749	12.66 ± 0.12	6.536 ± 0.108	774.6 ± 31.35
IV	4.2 ± 0.988	14.12 ± 0.443	7.03 ± 0.225	798.2 ± 22.891
V	3.14 ± 0.486	11.54 ± 0.331^∗∗^	5.63 ± 0.215^∗∗^	559.6 ± 71.08
II	3.88 ± 0.939	13.6 ± 0.391	6.774 ± 0.177	915 ± 42.588
VI	4.44 ± 0.736	12.6 ± 0.217	6.344 ± 0.031	857.4 ± 74.41

*Note:* Values are expressed as mean ± SEM. ^∗^*p* < 0.05, ^∗∗^*p* < 0.01, ^∗∗∗^*p* < 0.001, and ^∗∗∗∗^*p* < 0.0001 when compared with Group I. Groups II and VI are recovery groups. ^∗^*p* < 0.05, ^∗∗^*p* < 0.01, ^∗∗∗^*p* < 0.001, and ^∗∗∗∗^*p* < 0.0001 when compared with Group II.

**Table 4:** Serum biochemical parameters in male Wistar rats in various groups.

Groups	Glucose (mg/dL)	TC (mg/dL)	TG (mg/dL)	HDL (mg/dL)	AST (U/L)	ALT (U/L)	ALP (U/L)	TP (g/dL)	Albumin (g/dL)	Bilirubin total (mg/dL)	Bilirubin direct (mg/dL)	Urea (mg/dL)	Creatine (mg/dL)	Uric acid (mg/dL)
I	113.72 ± 7.65	115.34 ± 3.19	66.8 ± 2.61	40.42 ± 2.95	117.30 ± 4.25	68.84 ± 3.33	187.34 ± 7.77	7.35 ± 0.17	4.70 ± 0.08	0.42 ± 0.02	0.22 ± 0.02	35.2 ± 0.98	0.22 ± 0.02	3.34 ± 0.17
III	98.6 ± 4.83	123.96 ± 1.90	56.54 ± 1.41	44.00 ± 1.7	118.22 ± 4.77	78.52 ± 3.36	179.57 ± 9.91	7.58 ± 0.11	4.60 ± 0.11	0.5 ± 0.03	0.32 ± 0.04	39.94 ± 2.32	0.26 ± 0.02	3.35 ± 0.16
IV	112.68 ± 3.31	122.80 ± 6.45	62.9 ± 7.93	45.42 ± 3.71	113.00 ± 9.95	67.98 ± 3.98	207.78 ± 34.68	7.65 ± 0.25	4.58 ± 0.17	0.48 ± 0.03	0.26 ± 0.02	38.32 ± 0.28	0.26 ± 0.01	2.85 ± 0.33
V	105.92 ± 9.86	123.88 ± 2.40	51.06 ± 4.11	32.44 ± 1.01	103.66 ± 6.61	57.86 ± 0.81	137.64 ± 7.19	7.40 ± 0.17	4.50 ± 0.10	0.5 ± 0.03	0.26 ± 0.02	44.4 ± 1.01^∗∗∗^	0.23 ± 0.01	4.09 ± 0.56
II	112.09 ± 4.04	121.4 ± 1.64	62.1 ± 4.95	31.84 ± 0.99	119.76 ± 7.68	67.32 ± 2.83	161.55 ± 18.72	7.75 ± 0.24	4.18 ± 0.11	0.30 ± 0.03	0.14 ± 0.02	42.52 ± 1.89	0.18 ± 0.01	4.93 ± 0.75
VI	117.22 ± 7.78	120.04 ± 2.86	62.2 ± 6.66	30.42 ± 1.98	124.00 ± 2.74	75.76 ± 4.44	173.95 ± 23.56	7.62 ± 0.11	4.12 ± 0.05	0.36 ± 0.02	0.1 ± 0.00	43.54 ± 2.74	0.14 ± 0.02	5.34 ± 0.73

*Note:* Values are expressed as mean ± SEM. ^∗^*p* < 0.05, ^∗∗^*p* < 0.01, ^∗∗∗^*p* < 0.001, and ^∗∗∗∗^*p* < 0.0001 when compared with Group I. Groups II and IV are recovery groups. ^∗^*p* < 0.05, ^∗∗^*p* < 0.01, ^∗∗∗^*p* < 0.001, and ^∗∗∗∗^*p* < 0.0001 when compared with Group II.

**Table 5:** Serum biochemical parameters in female Wistar rats in various groups.

Groups	Glucose (mg/dL)	TC (mg/dL)	TG (mg/dL)	HDL (mg/dL)	AST (U/L)	ALT (U/L)	ALP (U/L)	TP (g/dL)	Albumin (g/dL)	Bilirubin total (mg/dL)	Bilirubin direct (mg/dL)	Urea (mg/dL)	Creatine (mg/dL)	Uric acid (mg/dL)
I	119.5 ± 8.03	99.72 ± 12.11	67.18 ± 4.43	42.14 ± 0.47	122.54 ± 6.45	59.78 ± 4.18	113.94 ± 12.50	7.31 ± 0.17	4.26 ± 0.1	0.38 ± 0.04	0.22 ± 0.04	35.00 ± 3.91	0.27 ± 0.01	3.01 ± 0.18
III	117.67 ± 10.06	126.50 ± 11.59	52.12 ± 2.13	46.2 ± 3.26	129.58 ± 19.89	51.3 ± 2.65	121.69 ± 7.84	7.56 ± 0.58	4.16 ± 0.16	0.4 ± 0.07	0.2 ± 0.03	37.44 ± 2.72	0.29 ± 0.01	3.94 ± 0.69
IV	99.5 ± 6.45	105.71 ± 4.55	48.72 ± 1.59	40.82 ± 0.63	107.75 ± 4.08	51.5 ± 1.03	111.85 ± 11.23	7.14 ± 0.02	4.28 ± 0.03	0.5 ± 0.04	0.28 ± 0.03	36.56 ± 0.14	0.32 ± 0.01	3.72 ± 0.34
V	120.28 ± 14.12	129.72 ± 3.87	56.00 ± 1.53	48.12 ± 2.12	131.54 ± 7.57	66.12 ± 11.59	136.89 ± 19.56	7.50 ± 0.18	4.56 ± 0.02	0.5 ± 0.04	0.22 ± 0.02	55.42 ± 3.78^∗∗∗^	0.36 ± 0.02^∗∗^	6.08 ± 0.75^∗∗^
II	111.42 ± 6.008	124.5 ± 8.55	75.18 ± 7.98	34.78 ± 2.96	183 ± 21.97	59.64 ± 4.16	121.69 ± 7.84	7.79 ± 0.46	4.5 ± 0.3	0.38 ± 0.08	0.2 ± 0.05	37.18 ± 1.38	0.18 ± 0.01	5.33 ± 0.76
VI	131.70 ± 6.35	126.6 ± 9.09	78.98 ± 4.43	39.26 ± 3.88	166.82 ± 23.1	59.62 ± 2.01	144.04 ± 18.77	7.85 ± 0.41	3.94 ± 0.12	0.4 ± 0.03	0.18 ± 0.03	42.65 ± 3.14	0.172 ± 0.01	5.078 ± 0.03

*Note:* Values are expressed as mean ± SEM. ^∗^*p* < 0.05, ^∗∗^*p* < 0.01, ^∗∗∗^*p* < 0.001, and ^∗∗∗∗^*p* < 0.0001 when compared with Group I. Groups II and VI are recovery groups. Values are expressed as mean ± SEM. ^∗^*p* < 0.05, ^∗∗^*p* < 0.01, ^∗∗∗^*p* < 0.001, and ^∗∗∗∗^*p* < 0.0001 when compared with Group II.

## Data Availability

The data are available on request due to privacy/ethical restrictions.

## Supporting Information

Supporting Information 1 Supporting Table 1: Details of composition of the test compound—Ayurvedic dentifrice used in this study for toxicity testing.

Supporting Information 2 ARRIVE Checklist: Adherence of the animal study to ARRIVE guidelines as per the checklist provided is counterchecked with the present study.
