# Supplementary material for: Preclinical Safety Assessment of an Ayurvedic Dentifrice: Acute and 28-Day Oral Toxicity Studies in Wistar Albino Rats
Source: J Toxicol. 2025 Aug 26;2025:1379571. doi: 10.1155/jt/1379571 (PMC12404827; doi:10.1155/jt/1379571)
Supplement: Supporting Information 1 — Supporting Table 1: Details of composition of the test compound—Ayurvedic dentifrice used in this study for toxicity testing. [file 1379571.f1.docx]

**SUPPLEMENTARY TABLE – 1**

1. Miswak (Salvadora Persica)

2. Licorice – Glycyrrhiza Glabra

3. Chebulic Myrobalan

4. Beleric Myrobalan

5. Dry ginger – Zingiber Officinale

6. Honey

7. Virgin coconut oil

8. Aloe Vera Juice

9. Black Pepper

10. Clove Bud

11. Cardamom

12. Cinnamon

13. Eucalyptus Oil

14. Peppermint

15. Rosemary Officinalis

16. Neem

17. Turmeric
